# MMR Vaccination and Hen’s Egg Allergy: Bridging the Gap Between Evidence and Clinical Practice

**DOI:** 10.3390/vaccines14060511

**Published:** 2026-06-05

**Authors:** Weronika Marta Balas, Maja Kaczor, Joanna Strzelecka, Adam Jerzy Sybilski

**Affiliations:** 1 Clinical Department of Pediatrics and Allergology, National Medical Institute of the Ministry of the Interior and Administration, 02-507 Warsaw, Poland; maja.kaczor03@gmail.com (M.K.); adam.sybilski@pimmswia.gov.pl (A.J.S.); 2 Department of Pediatrics, Centre of Postgraduate Medical Education, 01-813 Warsaw, Poland

**Keywords:** immunisation safety, food allergy, vaccine hesitancy, paediatric allergy, anaphylaxis risk

## Abstract

**Background**: Measles has re-emerged in recent years as a public health concern in the context of insufficient vaccination coverage. Some children experience significant delays in receiving, or refuse to take, the measles, mumps and rubella (MMR) vaccination, often due to concerns related to hen’s egg allergy (HEA). **Methods**: In this study, we retrospectively assessed the safety of MMR vaccination (Priorix^®^, GlaxoSmithKline, Belgium) in patients with HEA hospitalised at our clinic. Detailed medical histories were collected, along with skin prick tests and measurements of specific IgE against milk and egg proteins or extracts. The study included 39 patients with a mean age of 19 months, of whom 15 had previously experienced an anaphylactic reaction after egg ingestion. **Results**: None of these patients experienced a systemic reaction to vaccination. One patient developed a generalised maculopapular rash, which resolved after a single dose of an antihistamine. Vaccination was postponed in 63% of patients, with the longest delay extending to 113 months. **Conclusions**: Severe adverse reactions following MMR vaccination in patients with HEA are generally rare and are outweighed by the risks associated with natural infection and its complications. Effective communication of vaccine safety data and strengthening public trust in healthcare professionals are crucial.

## 1. Introduction

Vaccinations are considered safe for the vast majority of patients [1]. The existing literature suggests that parental concerns about potential adverse and hypersensitivity reactions are among the predominant factors contributing to hesitancy toward childhood vaccines [1]. Parents often question the appropriateness of immunisation in children with hen’s egg allergy (HEA) and are concerned about allergic reactions. Studies conducted in Europe indicate that as many as 18.71% of parents believe their child has a food allergy [2]. However, epidemiological data estimate the prevalence of food allergy at approximately 6–8% at the age of two years and 3–4% during adolescence [3]. The most common causes of food allergy in children are allergens derived from cow’s milk (CM) and hen’s egg (HE) [4]. One of the vaccines most commonly—but incorrectly—associated with the risk of allergic reactions in patients with HEA is the measles, mumps and rubella (MMR) vaccine. The MMR vaccine contains live attenuated MMR viruses. MMR vaccination in Poland is mandatory and provided free of charge within the national immunisation program, with the first dose administered at 13–15 months of age and second dose at 6 years of age. MMR viruses are propagated in chick embryo fibroblast cell cultures and do not contain egg proteins in clinically significant amounts. However, isolated reports have suggested a possible association between higher levels of HE-specific IgE (sIgE) and the occurrence of symptoms following vaccination [5], although this finding has not been replicated in larger studies. The overall vaccine safety profile in Poland is reassuring: approximately 0.05% of all administered doses are associated with adverse events following immunisation (AEFIs), of which approximately 90% reported after Priorix^®^ were mild, predominantly local reactions and fever [6]. Despite substantial evidence confirming the safety of MMR vaccines in individuals with HEA, patients with a history of HEA are still frequently referred for vaccination in hospital settings [5]. This practice is associated with increased healthcare costs, delays in vaccination, unnecessary exposure to hospital pathogens, and reduced bed availability for children with urgent needs. The aim of this study was to assess the safety of MMR vaccination in children with a history of HEA and to evaluate the discrepancy between evidence-based recommendations and clinical practice.

## 2. Materials and Methods

A detailed retrospective analysis was conducted of the digital hospital records of patients with a positive history of HEA who received MMR vaccination (Priorix^®^, GlaxoSmithKline, Rixensart, Belgium) during hospitalisation at the Department of Pediatrics and Allergology of the Central Clinical Hospital of the Ministry of the Interior and Administration (PIM MSWiA) in Warsaw, Poland, between January 2021 and March 2026. It was designed as a retrospective cohort study. Personal data of study participants were fully anonymised prior to statistical analysis to ensure that individual patients could not be identified. The following variables were analysed: age, sex, place of residence (urban vs. rural), results of food allergy panels and molecular diagnostics (Macro Array Diagnostics (MADX) Austria, Wien; ImmunoCAP^TM^ and ImmunoCAP^TM^ ISAC (Thermo Fisher Scientific Inc., Uppsala, Sweden) for CM proteins (Bos d 4, Bos d 5, Bos d 8, Bos d 6) and HE proteins (Gal d 1, Gal d 2), family history of allergic diseases, and the presence of atopic conditions (allergic rhinitis, asthma, atopic dermatitis, anaphylaxis and cow’s milk allergy). In addition, the occurrence of anaphylactic reactions was recorded, together with information on whether the patient was under the specialist care of an allergist and the daily use of anti-allergic medications. The inclusion criteria comprised children aged 18 years or younger with HEA who received the MMR vaccine during hospitalisation. HEA was defined by a positive oral food challenge (OFC) to HE. Additional diagnostic tests, such as skin prick tests and component-resolved diagnostics, were collected when available but were not mandatory for inclusion. The observation period covered January 2021 to February 2026. The indication for hospitalisation was the administration of MMR vaccination under conditions of increased precaution, with supervision by medical personnel experienced in recognising and managing severe hypersensitivity reactions. Patient observation ended at the time of hospital discharge, at least 4 h after vaccination. During the post-vaccination period, blood pressure, heart rate, and saturation were monitored, along with close observation of the child by a nurse. Patients were excluded if clinical data were insufficient to diagnose HEA in children, including the absence of an oral food challenge.

### 2.1. Data Analysis

Only descriptive statistical analyses were performed for all study variables. Continuous variables, such as age, were presented as means ± standard deviations for normally distributed data or as medians with interquartile ranges for non-normally distributed data. Categorical variables were summarised as absolute numbers and percentages.

### 2.2. Institutional Review Board Statement

The study protocol received approval from the Bioethics Committee of the National Medical Institute of the Ministry of the Interior and Administration in Warsaw under number 70/2026 on 11 March 2026. Informed consent was not required due to the retrospective nature of the study and the anonymization of the data.

## 3. Results

Between 2021 and 2026, a total of 39 MMR vaccinations were performed in our department, none of which resulted in a systemic reaction. Baseline characteristics of the study population are presented in Table 1. Mean age was 19 months (IQR: 14.25–26.75) and average was 30.45 ± 29.31 months. All patients had a previously established diagnosis of HEA confirmed by a positive OFC. Additional diagnostic tests are presented in Table 1. No patient underwent multiple diagnostic modalities. In this cohort, 38.46% of children had previously experienced an anaphylactic reaction following HE ingestion. None of these patients developed an anaphylactic reaction after receiving the MMR vaccine.

In our study, only one patient developed symptoms—a generalised maculopapular rash—which resolved after a single standard dose of an antihistamine.

## 4. Discussion

Family physicians, paediatricians, and even allergists continue to refer children with HEA for MMR vaccination under enhanced medical supervision. According to current guidelines from the European Academy of Allergy and Clinical Immunology, children with HEA can safely receive the MMR vaccine without the need for enhanced clinical supervision, as currently available MMR vaccines do not contain ovalbumin in clinically relevant quantities [7]. This issue was already investigated between 1982 and 1996, when the incidence of adverse reactions following MMR vaccination was shown to be extremely low: on average, per 100,000 administered doses, only one patient developed anaphylaxis, one experienced urticaria, and 0.3 patients developed asthma [8]. With advances in technology, ex vivo studies have further clarified the underlying pathophysiology, demonstrating the absence of vaccine-specific B-cell proliferation following exposure to the MMR vaccine in a patient with HEA [9]. Moreover, subsequent reports have indicated that anaphylaxis after MMR vaccination is not attributable to an IgE-mediated reaction to HE proteins, but rather to an IgE-mediated response to gelatin or neomycin, a stabiliser present in high concentration in the vaccine formulation [9]. We suspect that the reaction in our patient may have been related to other vaccine components. Furthermore, an alpha-gal syndrome can be another reason for anaphylaxis following MMR vaccination [10]. In previous MMR vaccines, dextran was used as a stabiliser. However, once it was shown to activate IgG and the complement system, thereby inducing allergic reactions, it was subsequently withdrawn from the market [11]. The reported incidence of anaphylaxis following MMR vaccination is 5.14 per million doses, whereas common minor local reactions occur in 1 in 20 doses [7]. The existing literature shows that the risk of hypersensitivity reactions following MMR vaccination in patients with HEA is comparable to that observed in the general population [7]. Children with HEA should receive routine MMR vaccination in primary care. Only a severe hypersensitivity reaction following a previous dose of MMR vaccine—regardless of the suspected component—constitutes a permanent contraindication to further administration [8]. Nevertheless, one study has suggested that HE-sIgE thresholds might predict risk following MMR vaccination [5]. The finding, based on a small cohort, is hypothesis-generating. However, this kind of predictive biomarker has not yet been widely validated or incorporated into clinical guidelines.

Despite numerous reports confirming its safety, parents still encounter refusal of vaccination in outpatient settings. According to reports from the European Centre for Disease Prevention and Control, the incidence of measles has increased markedly in recent years, while most countries continue to have insufficient vaccination coverage. In 2022, only four countries in the European Union achieved the recommended vaccination coverage of at least 95% (with two doses) [12]. An increasing number of unvaccinated individuals, including young children, only serves to exacerbate this trend, leading to a wider spread of measles and a greater risk of severe complications associated with the disease. Concerns surrounding vaccination resulted in the postponement of immunisation in 63% of our patients, with the longest delay extending to 113 months, which largely accounts for the highly heterogeneous age distribution observed in our cohort. These findings highlight the importance of effective dissemination of vaccine safety data and strengthening public trust in healthcare professionals. Improving communication strategies may help address vaccine hesitancy. Achieving high vaccination coverage is essential, particularly as the number of children migrating due to armed conflicts continues to increase. Due to migration, many children have limited access to vaccinations or have not received them as a result of war. In addition, there remains a group of individuals who deliberately avoid vaccination. In many cases, parents lack clear, evidence-based information and remain concerned due to the persistent myth linking HEA with MMR vaccine safety, a gap not always effectively addressed by healthcare professionals. In our experience, some physicians still recommend HE exposure prior to vaccination to assess anaphylaxis risk. Improving vaccination coverage requires a multilevel approach, combining individual strategies (education, reminders, provider interventions) with public measures (financial incentives, policy, legislation), as only multicomponent interventions have proven effectiveness [13]. This descriptive observational study has inherent limitations, including its small sample size and retrospective design. Nevertheless, it sheds light on an issue of considerable and immediate relevance to contemporary medical practice and public health. At a time of rising measles incidence and persistent vaccine hesitancy across Europe, evidence clarifying the safety of MMR vaccination in children with HEA carries direct implications for vaccination policy, primary care practice, and public health strategy.

## 5. Conclusions

The existing literature consistently shows that adverse events following MMR vaccination are rare, while the risks of measles infection and its associated complications are substantially higher. In light of the increasing incidence of measles in Europe, we address this important public health issue. Therefore, the implementation of multilevel, multicomponent interventions targeting both parents and healthcare professionals is essential.

## Figures and Tables

**Table 1:** General characterisation of the study group.

	Variable	n (%)
	Total	39 (100%)
Demographics	Female sex	17 (43.59)
	Male sex	22 (56.41)
	Place of residence—City	33 (84.62)
	Place of residence—Rural	6 (15.38)
Type of allergy test performed	ALEX	18 (46.15)
	single ImmunoCAP	9 (23.08)
	ISAC	4 (10.26)
	Skin prick test	1 (2.56)
	Not available	7 (17.95)
Laboratory allergy test—HE	HE white protein (positive)	23 (58.97)
	HE yolk (positive)	12 (30.77)
	Ovalbumin (positive)	21 (53.85)
	Ovomucoid (positive)	17 (43.59)
Laboratory allergy test—CM	CM (positive)	13 (33.33)
	Bos d 4 (positive)	7 (17.95)
	Bos d 5 (positive)	8 (20.51)
	Bos d 8 (positive)	11 (28.21)
Skin prick test	HE (positive)	1 (2.56)
Clinical characteristics	Family history of allergic diseases	17 (43.59)
	Specialist allergy care	34 (87.18)
	Regular use of anti-allergic medication	13 (33.33)
Comorbidities	Allergic rhinitis	9 (23.08)
	Asthma	11 (28.21)
	Atopic dermatitis	19 (48.72)
	Anaphylaxis	15 (38.46)
	Cow’s milk allergy	19 (48.72)

HE—hen’s egg; CM—cow’s milk.

## Data Availability

The raw data supporting the conclusions of this article will be made available by the authors upon request, while maintaining the anonymity of the patients.
